# Investigation of Efficient Mixing Enhancement in a Droplet Micromixer with Short Mixing Length at Low Reynolds Number

**DOI:** 10.3390/mi16060715

**Published:** 2025-06-16

**Authors:** Yuanfang Qiu, Xueze Zhang, Mengzhen Hao, Xu Yin, Mengling Zhou, Shichao Ma, Yuanting Zhang, Naiqian Jiang, Li Xie, Xichen Yuan, Honglong Chang

**Affiliations:** 1 School of Mechanical Engineering, Northwestern Polytechnical University, Xi’an 710072, China; qyf@yxopt.com (Y.Q.); zhangxueze@mail.nwpu.edu.cn (X.Z.); mengzhenhao@mail.nwpu.edu.cn (M.H.); yinxuu@mail.nwpu.edu.cn (X.Y.); zhoumengling233@163.com (M.Z.); msc-2021@mail.nwpu.edu.cn (S.M.); zhangyt612@mail.nwpu.edu.cn (Y.Z.); jiangnaiqian@mail.nwpu.edu.cn (N.J.); 2 Ministry of Education Key Laboratory of Micro and Nano Systems for Aerospace, School of Mechanical Engineering, Northwestern Polytechnical University, Xi’an 710072, China; 3 Ningbo Yongxin Optics Co., Ltd., 385 Mingzhu Road, Hi-Tech Industry Park, Ningbo 315040, China; 4 Key Laboratory for Space Bioscience and Biotechnology, Northwestern Polytechnical University, Xi’an 710072, China; xieli@nwpu.edu.cn; 5 School of Life Sciences, Northwestern Polytechnical University, Xi’an 710072, China

**Keywords:** rapid mixing, Taylor flow, chaotic convection, serpentine microchannels, low Reynolds number

## Abstract

Rapid mixing is widely prevalent in the field of microfluidics, encompassing applications such as biomedical diagnostics, drug delivery, chemical synthesis, and enzyme reactions. Mixing efficiency profoundly impacts the overall performance of these devices. However, at the micro-scale, the flow typically presents as laminar flow due to low Reynolds numbers, rendering rapid mixing challenging. Leveraging the vortices within a droplet of the Taylor flow and inducing chaotic convection within the droplet through serpentine channels can significantly enhance mixing efficiency. Based on this premise, we have developed a droplet micromixer that integrates the T-shaped channels required for generating Taylor flow and the serpentine channels required for inducing chaotic convection within the droplet. We determined the range of inlet liquid flow rate and gas pressure required to generate Taylor flow and conducted experimental investigations to examine the influence of the inlet conditions on droplet length, total flow rate, and mixing efficiency. Under conditions where channel dimensions and liquid flow rates are identical, Taylor flow achieves a nine-fold improvement in mixing efficiency compared to single-phase flow. At low Reynolds number (0.57 ≤ Re ≤ 1.05), the chip can achieve a 95% mixing efficiency within a 2 cm distance in just 0.5–0.8 s. The mixer proposed in this study offers the advantages of simplicity in manufacturing and ease of integration. It can be readily integrated into Lab-on-a-Chip devices to perform critical functions, including microfluidic switches, formation of nanocomposites, synthesis of oxides and adducts, velocity measurement, and supercritical fluid fractionation.

## 1. Introduction

Microfluidics is a rapidly developing and widely applied discipline. In the fast-evolving landscape of Lab-on-a-Chip (LOC) technology, microfluidic systems have found applications in various fields, including biomedical diagnostics, drug delivery, chemical synthesis, and enzyme reactions [1]. LOC devices integrate multiple functionalities within a chip, encompassing tasks such as mixing, reactions, separation, and analysis [2]. Micromixers, as crucial components of microfluidic systems, serve the purpose of blending two or more samples for subsequent processes [3]. The efficiency of mixing can directly impact the overall performance of the system [4]. However, achieving rapid mixing in microfluidics presents challenges due to the predominantly laminar flow at low Reynolds numbers within microchannels. Weak convective flow and high surface tension within the fluids limit convective mixing [5], relying predominantly on molecular diffusion [6], which is an inefficient mode of mixing [7]. This necessitates longer channels to achieve uniform mixing [8]. Based on existing research on micromixers, they can be categorized as active mixers and passive mixers, depending on whether external energy input is required [9].

Over the past three decades, microfluidic mixing has evolved through landmark designs, including active mixers (e.g., acoustic [10], magnetic [11], etc.) and passive mixers (e.g., lamination [12], chaotic convection [13], and droplet-based [14] methods). Active mixers, while effective, face limitations such as complex fabrication, external energy dependence, and potential damage to sensitive biomolecules [9]. Recent innovations in active mixing have demonstrated remarkable capabilities, particularly surface acoustic wave (SAW) technologies coupled with bubble-induced streaming. These hybrid approaches achieve mixing times under 100 ms through bubble oscillation and acoustic streaming synergies. However, they require complex transducer arrays and precise bubble size control [15], limiting their scalability compared to passive systems. Passive mixers, though simpler, often struggle to achieve rapid mixing at low Reynolds numbers (Re < 10) due to weak convective flows and reliance on molecular diffusion [5,6,7]. For instance, serial lamination mixers [16] perform well at moderate Re (10–70) but fail in low-Re regimes [17], while chaotic convection mixers [13] require intricate geometries prone to clogging [18]. Modern chaotic mixers have evolved beyond traditional geometries, incorporating 3D fractal structures that achieve 90% mixing within 5 mm at Re = 0.5 [19]. While effective, these designs often require advanced fabrication techniques like multi-layer lithography, presenting manufacturing challenges our planar design avoids. Droplet-based mixers [14,20,21] leverage Taylor flow vortices and chaotic advection, yet systematic studies on optimizing inlet conditions (e.g., flow rates, gas pressure) for low-Re applications remain scarce [22,23].

Despite these advances, key challenges persist: (1) achieving high mixing efficiency (>90%) at ultra-low Re (<5) without complex geometries or external energy inputs; (2) minimizing mixing length (<5 cm) and time (<1 s) for portable LOC devices; and (3) bridging the gap between theoretical models and practical, scalable designs. Recent work by Pinho et al. [24] highlights the potential of Dean-Taylor flows, but their focus on bi-phasic systems leaves room for optimizing single-phase-like droplet mixing. These challenges are particularly critical for three emerging applications: (1) point-of-care blood testing requiring gentle mixing of viscous whole blood (Re ≈ 1) in compact devices; (2) microfluidic protein synthesis where faster mixing prevents protein misfolding; (3) portable environmental monitoring needing <1 s mixing for rapid pollutant detection. 

Here, we address these gaps by integrating Taylor flow vortices with chaotic convection in serpentine channels, enabling rapid mixing (≥95% efficiency) within 2 cm and 0.5–0.8 s at Re = 0.57–1.05. Our design departs from prior work as follows: (1) by systematically mapping inlet conditions (liquid flow rate, gas pressure) to droplet length and mixing efficiency; (2) combining T-junction and serpentine geometries in a monolithic, clog-resistant PMMA chip; and (3) demonstrating performance superior to classic designs [22,25] and recent alternatives [24]. The mixer developed in this study, operating at low Reynolds numbers, is particularly suitable for applications requiring gentle fluid handling, such as point-of-care diagnostics (e.g., blood analysis), sensitive biochemical reactions, and microorganism cultivation. This Re range enables efficient mixing while minimizing shear stress, making it ideal for processing delicate biological samples and shear-sensitive fluids. The target performance thresholds (≥95% efficiency in 0.5–0.8 s) are derived from the following: (i) WHO ASSURED standards requiring <1 s mixing for point-of-care diagnostics [26], (ii) nanoparticle synthesis needing >90% efficiency for PDI <0.2 [27], and (iii) JIS K 0129 environmental protocols mandating 95% homogeneity [24]. This approach balances simplicity, scalability, and performance, making it ideal for low-Re applications in diagnostics and chemical synthesis.

## 2. Experimental Section

### 2.1. Micromixer Design

**Figure 1:**
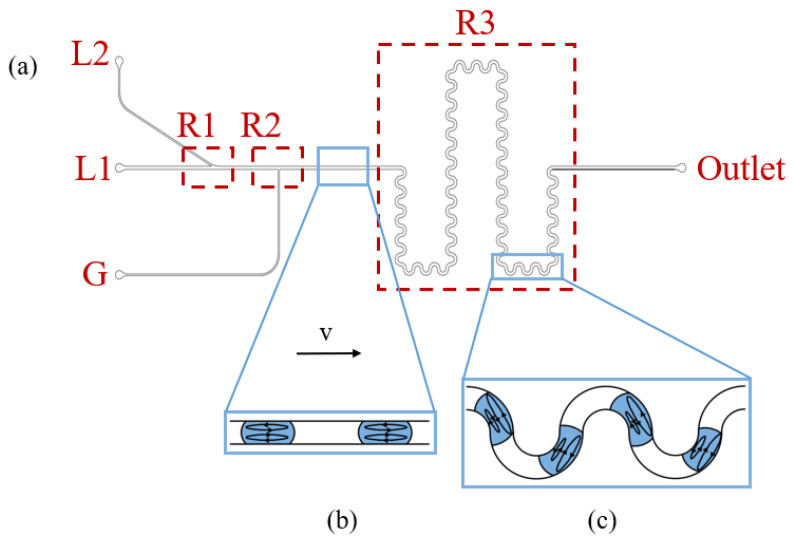
(**a**) Schematic diagram of the chip structure. The chip contains three inlets ($I_{L1}$, $I_{L2}$, and $I_{G}$) and one outlet, where the angle between $I_{L1}$ and $I_{L2}$ channels is 30° to realize laminar flow injection of the two liquids in the main channel corresponding to R1, while the $I_{G}$ channel is perpendicular to the main channel to generate Taylor flow corresponding to R2. The droplets generated by Taylor flow are mixed mainly through chaotic convection in serpentine channels (R3). (**b**) The vortices within the droplet are symmetrical when the droplets flow in a straight channel. (**c**) The vortices within the droplet become asymmetric, and their size and position change in accordance with the direction of curvature when the droplets flow in the serpentine channel.

The schematic diagram of the proposed micromixer structure is shown in Figure 1a. The chip consists of two liquid inlets ($I_{L1}$ and $I_{L2}$), one gas inlet ($I_{G}$), and one outlet. The primary structures include a Y-shaped liquid sample introduction region (R1) to realize laminar flow injection, a T-shaped Taylor flow generation region (R2), and a chaotic mixing region (R3). Specifically, the widths of the channel at the $I_{L1}$ and $I_{L2}$ are 300 µm and 150 µm, respectively, with an angle of 30° between the two liquid inlet channels [28], where the channel containing $I_{L1}$ is the main channel. The width of the channel containing the $I_{G}$ is 150 µm, and the curvature of the serpentine channels is set at 2 in R3 [29]. The depth of the overall channel across the chip is 150 µm.

The working principle of the micromixer is shown in Figure 1. Two fluids flow into the micromixer at R1 in parallel in the form of upper and lower laminar flows and are transformed into Taylor flow at R2. In Figure 1b,c, we illustrate the flow field distribution in the axial cross-section of a typical Taylor flow configuration. When the droplet moves in the straight channel, the liquid moves from the center of the channel, flows towards the channel wall due to the influence of the front interface, and the liquid of the droplet moves from the wall to the center due to the influence of the rear interface, resulting in a pair of symmetrical, circumferential and counter-rotating vortices within a moving discrete drop [30], as shown in Figure 1b. In the straight channel, mixing in the droplet under Taylor flow is dominated by diffusion across the interface of the two rotational vortices [24]. When the Taylor flow passes through the serpentine channel (R3), with the introduction of curvature, the vortices within the droplet become asymmetric, and their size and position change in accordance with the direction of curvature, as shown in Figure 1c, the use of serpentine microchannels further enhances the mixing efficiency of the liquid droplets [31].

### 2.2. Micromixer Fabrication

The chip consists of two 5 mm thick plates of polymethyl methacrylate (PMMA), substrate and cover, processed by Beijing Jingdiao Group (Xi’an, Shaanxi, China). As shown in Figure 1a, each channel was carved in the substrate and sealed with a cover. Firstly, two processed PMMA plates were immersed in IPA for ultrasonic cleaning for 5 min. Following this, they were rinsed for 30 s in deionized water, dried using nitrogen gas, and subjected to annealing in a convection oven. Finally, plasma treatment was applied to the PMMA plates, and the aligned PMMA plates were placed into a thermocompressor. The bonding process was executed at a temperature of 95 °C, a pressure of 2756 kPa, and maintained for 600 s. The chip was then naturally cooled to 40 °C, completing the chip bonding process.

### 2.3. Materials

To generate Taylor flow in this study, we introduced a 75% ethanol solution (viscosity coefficient µ_1_ = 2.0 mPa·s) and a 25% ethanol solution (viscosity coefficient µ_2_ = 2.4 mPa·s) as two liquid phases, and air was employed as the gas phase. To clearly show the mixing process in the microchannel, methylene blue dye ($C_{16}H_{18}{\mathrm{CIN}}_{3}S$) was added to the 25% ethanol solution, resulting in a blue coloration, where the concentration of methylene blue was 20 mg/mL, while the 75% ethanol solution did not contain any dye.

### 2.4. Experimental Setup

**Figure 2:**
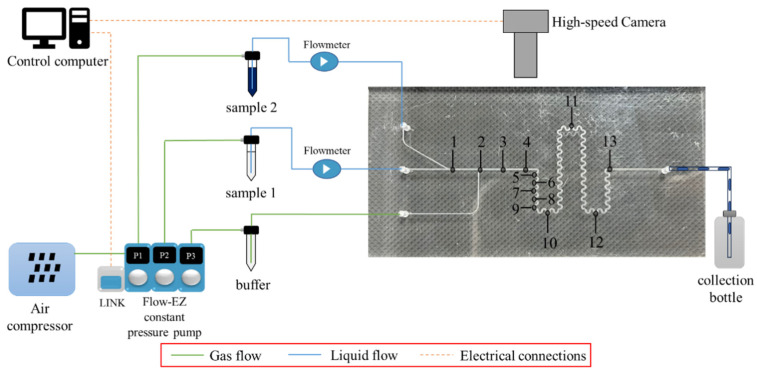
Experimental platform. The platform mainly contains three modules, which are the fluidic module, the optical module, and the control module. The fluidic module that injects liquid and gas phases into the chip to generate Taylor flow (point 2) comprises an air compressor, three constant pressure pumps, two flow sensors, and the mixing chip. The optical module comprises an optical microscope and a high-speed CCD camera to observe the generation of the Taylor flow and the mixing of different liquids. The control module is primarily operated by a computer to observe, adjust, and record the inlet conditions and mixing images.

The experimental platform of the mixing chip based on Taylor flow and chaotic convection is shown in Figure 2, which can be divided into the fluidic module, optical module, and control module. The fluidic module comprises an air compressor (TYW-500, XRS, Beijing Xiruns Instrument Co., Ltd., Beijing, China), three constant pressure pumps (FLOW-EZ, Fluigent, Le Kremlin-Bicêtre, France, adjustable range is 0–1000 mbar), two flow sensors (FLOW UNIT, Fluigent, France, the accuracy is 7 nL/min), and the mixer. Initially, the air compressor provides pressure to constant pressure pumps, which deliver a constant flow of the 75% ethanol solution and the 25% ethanol solution from the respective reservoirs into the mixing chip through $I_{L1}$ and $I_{L2}$. The flow rate ratio is maintained at 5.5:1, i.e., the ratio of $Q_{l1}$ to $Q_{l2}$ is 5.5:1. Air is introduced into the mixer via $I_{G}$ using a constant-pressure pump. To control the precision of the inlet flow rates of the liquid reagents, flow sensors are connected between the liquid reagent reservoirs and the chip. Additionally, Luer tapers are used to establish connections between the reservoirs and flow sensors, while steel needles are employed for connecting the tubing to the mixer.

The optical module includes an optical microscope and a high-speed CCD camera (VEO-E 340L, Phantom, Vision Research, Inc., Wayne, NJ, USA), where the camera’s pixel depth is set at 8 bits having 256 gray scale. Since the small channel size and high Taylor flow rate, an optical microscope and a high-speed CCD camera are necessary to observe the generation of the Taylor flow in the microchannels and the mixing of different liquids. The entire flow channel cannot be observed due to the limited field of view of the microscope, so observation points needed to be arranged along the chip’s channel, as shown in Figure 2. Thirteen observation points are arranged on the mixer, with point 1 dedicated to observing laminar flow during the introduction of liquids, point 2 used to observe the generation of the Taylor flow when gas is introduced into the main channel, points 3–4 designated for observing the mixing of the Taylor flow within the straight channel, and points 5–13 designated for observing the mixing of the Taylor flow within the serpentine channels. Among them, in observation points 2–4, the distance between adjacent points is 6 mm, the distance between observation points 4 and 5 is 1.35 mm, and in observation points 5–8, the distance between adjacent points is 1.8 mm.

The control module is primarily operated by a computer. The constant pressure pumps are equipped with a LINK module for system control and direct communication with the computer. Through the A-i-O software, adjustments to the inlet flow rates and the inlet air pressure can be made. Additionally, real-time pressure and flow fluctuations within the channels can be observed and recorded. Furthermore, the computer communicates with the high-speed camera, where relevant parameters are configured within the PCC software (Phantom Camera Control_3.3.781.0, Vision Research, Inc., Wayne, NJ, USA) for image acquisition, enabling the recording of mixed images at each observation point.

**Figure 3:**
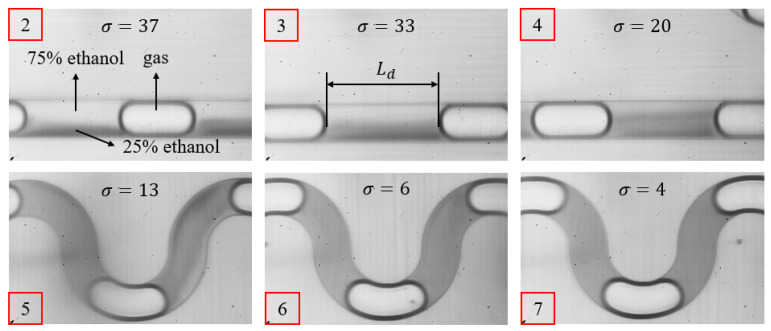
The mixing condition in the droplet at observation points 2–7. Injecting undyed 75% ethanol solution, dyed 25% ethanol solution, and air into the mixer generates Taylor flow, where the length of the droplet is $L_{d}$. The standard deviation of the gray scale value gradually decreased as the value of the observation point increased. The liquids in the droplet completed mixing at observation point 7.

By controlling the liquid flow rates and gas pressure, we successfully generated Taylor flow, as shown in Figure 3, which shows the Taylor flow image from point 2 to point 7. Here, $L_{d}$ represents the length of the droplet. Automated programs using NI Vision Assistant in LabVIEW are programmed to postprocess and analyze the images to extract data associated with droplet length and droplet gray scale standard deviation (details are provided in the Supplementary Information). We can determine the mixing efficiency using the following equation:

$$e_{mix}=\left(1-\frac{\sigma }{\sigma_{0}}\right)\times 100\%$$

In the equation, σ is the standard deviation of the gray scale value of the image captured at each observation point, and $\sigma_{0}$ is the standard deviation of the gray scale value in the initial unmixed state. Under this experimental condition, the fixed value of $\sigma_{0}$ is 80. Ideally, σ finally approaches 0 when the fluids are homogeneously mixed. Therefore, the mixing efficiency, denoted as $e_{mix}$, ranges from 0 to 100%, where a higher $e_{mix}$ implies more uniform mixing. In the experimental measurement, it is considered that an ideal homogeneous state has reached when $e_{mix}$ ≥ 95%. In Figure 3, as the droplets flow through the channel, the standard deviation of the gray scale value gradually decreases until the standard deviation is 4 at the observation point 7, where the liquids in the droplet can be regarded as completely mixed.

## 3. Results and Discussion

According to the research of Peng et al., the length of the droplets ($L_{d}$) and the total flow rate ($Q_{tot}=Q_{l}+Q_{g}$) jointly determine the mixing characteristics inside the droplet [23]. Among these factors, the droplet length is influenced by the injection conditions, liquid viscosity, channel geometry, and surface properties [32]. For this study, liquid viscosity, channel geometry, and surface properties were assumed to be fixed, so this paper primarily investigated the influence of gas–liquid injection conditions on droplet length and total flow rate. We initially determined the range of liquid flow rate and gas pressure for generating Taylor flow and compared the mixing efficiency between Taylor flow and single-phase flow in the mixer. Additionally, we conducted experimental studies to investigate the impact of liquid flow rate and gas pressure on droplet length, total flow rate, and mixing efficiency.

### 3.1. Define the Range of Liquid Flow Rate and Gas Pressure to Generate Taylor Flow

The ability to generate Taylor flow is a prerequisite for achieving efficient mixing in the mixer. Controlling the liquid and gas within the microchannel is critical for generating Taylor flow. Therefore, it is necessary to study the range of inlet liquid flow rate and gas pressure for generating Taylor flow.

**Figure 4:**
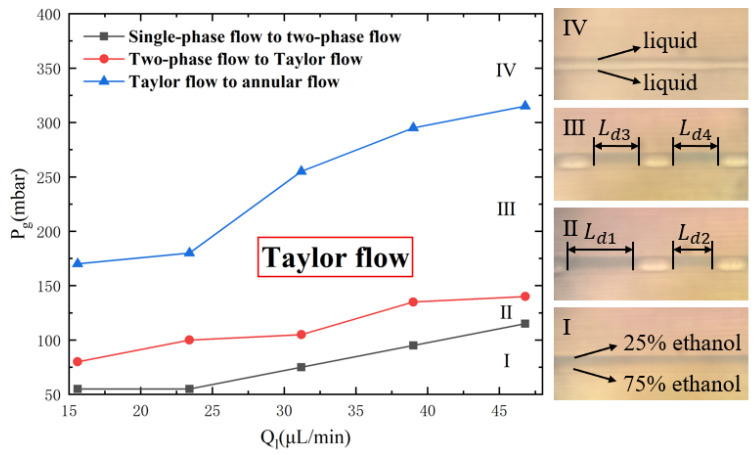
Flow pattern transition diagram. Stage I is called the Single-phase Flow Stage due to insufficient gas pressure. There is only liquid in the channel, the upper blue liquid is a 25% ethanol solution, and the lower liquid is a 75% ethanol solution. Stage II is called the Two-phase Flow Stage, and can generate the unstable two-phase flow ($L_{d1}>L_{d2}$) instead of the Taylor flow. Stage III is called the Taylor Flow Stage, and can generate the stable two-phase ($L_{d3}=L_{d4}$) known as the Taylor flow. Stage IV is called the Annular Flow Stage, due to excessive air pressure, which occurs when the phenomenon of the gas flows out from the center of the channel, and the liquid attaches to the wall of the channel.

According to the research by Günther et al. [33], when the apparent flow velocity of liquid ($v_{l}$) is 0.001~0.02 m/s, and the apparent flow velocity of gas ($v_{g}$) is 0.001~0.1 m/s, mixing occurs inside the droplet. Based on the cross-sectional area of the chip’s channels (A=300 μm×150 μm=45,000 μm^2^), the corresponding flow rates calculated from the equation Q=v×A are $Q_{l}$= 2.7–54 µL/min and $Q_{g}=2.7-270\;µL/\min$. The adjustment range of the total liquid inlet flow rate during the experiment was set to be 0–50 µL/min and the adjustment range of the gas pressure is 0–1000 mbar. Upon summarizing the preliminary experimental results, it was observed that as the total liquid flow rate and gas pressure changed, the flow pattern in the microchannel changed in four stages, as depicted in Figure 4.

Stage I: Single-phase Flow Stage. At this stage, the gas pressure is insufficient, and the liquid flows into the gas inlet channel. The input conditions can only generate single-phase flow and cannot generate Taylor flow. There are only liquids in the channel, as shown in Figure 4, the upper blue liquid is a 25% ethanol solution, and the lower liquid is a 75% ethanol solution.

Stage II: Two-phase Flow Stage. At this stage, the gas pressure is insufficient to establish a stable Taylor flow, and it only restrains the liquid flow at the mouth of the T-shaped channel. The resulting flow pattern of stage II is like that of stage III, but the two-phase flow exhibits an unstable state, producing droplets of varying lengths, as can be clearly seen from Figure 4, $L_{d1}>L_{d2}$.

Stage III: Taylor Flow Stage. At this stage, the gas pressure is approximately balanced with the liquid flow resistance. The Taylor flow is relatively stable, and the lengths of droplets are generally uniform, as can be clearly seen from Figure 4, $L_{d3}=L_{d4}$. Effective control of droplet length can be achieved by adjusting the gas pressure and liquid flow rate.

Stage IV: Annular Flow Stage. At this stage, further increasing the gas pressure causes a transition from Taylor flow to annular flow, where the gas flows out from the center of the channel, and the liquid adheres to the wall of the channel.

The transition from unstable two-phase flow (Stage II) to the stable Taylor flow (Stage III) was determined by two quantitative metrics: the droplet length variation coefficient (Cv): Stage II: Cv > 15% (non-uniform droplets); Stage III: Cv ≤ 10% (stable Taylor flow) and pressure fluctuation amplitude: Stage II: ΔP/Pavg > 8%; Stage III: ΔP/Pavg ≤ 5%. During the generation of the Taylor flow, both the liquid flow rate and gas pressure have an impact on the length of the droplet, affecting the mixing efficiency. Therefore, experiments were designed to investigate the influence of liquid flow rate and gas pressure on mixing efficiency.

### 3.2. The Comparison of Mixing Efficiency Between Taylor Flow and Single-Phase Flow

**Figure 5:**
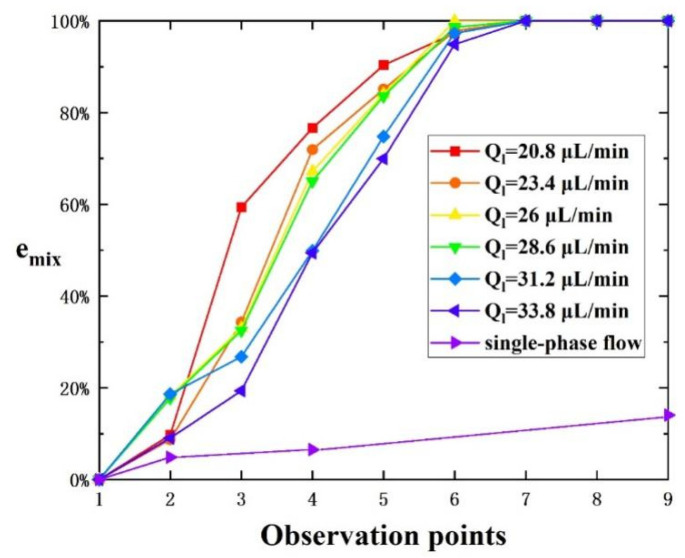
Mixing efficiency of observation points. It was obvious that the mixing efficiency of the Taylor flow at any inlet total liquid flow rate was higher than that of single-phase flow. And the droplet generated by Taylor flow achieved complete mixing at observation point 7, while the mixing efficiency of single-phase flow at observation point 7 was less than 20%.

While the low Re suggests laminar flow, established mechanisms enable chaotic convection: (1) curvature-induced Dean vortices, (2) periodic perturbation from serpentine turns, and (3) droplet-internal vortex asymmetry [34]. These collectively produce Lagrangian chaos as evidenced by our mixing efficiency (9× straight channel), matching theoretical predictions for chaotic advection. As shown in Figure 4, when the flow pattern in the microchannel was in Stage I, the gas pressure was relatively low, and the gas could not enter the main channel of the mixer, resulting in single-phase flow inside the microchannel. With the continuous increase in gas pressure, when the gas–liquid injection conditions were in Stage III, the Taylor flow was generated within the microchannel. At an inlet gas pressure of 110 mbar, the Taylor flow was generated at various inlet total liquid flow rates of 20.8 µL/min, 23.4 µL/min, 26 µL/min, 28.6 µL/min, 31.2 µL/min, and 33.8 µL/min. The corresponding Reynolds number Re, ranged from 0.57 to 1.05, calculated from Re=ρUDh/μ, where ρ is density, U is the average velocity, $D_{h}$ is the hydraulic diameter, μ is dynamic viscosity. The mixing efficiency of the Taylor flow and single-phase flow at $Q_{l}=26\;\mu L/\min$ at observation points 1–9 is shown in Figure 5. It was evident that the droplet generated by the Taylor flow at any inlet liquid flow rate achieved complete mixing at observation point 7, that is, the distance required for the droplet to achieve complete mixing from the generation of the Taylor flow at observation point 2 was not more than 2 cm. While the mixing efficiency of single-phase flow at observation point 7 was less than 20% and the single-phase flow was not fully mixed at observation point 13 (i.e., the end of the channel). The observation point 4 is located at the end of the straight channel, where the mixing efficiency of the Taylor flow at an inlet liquid flow rate of 26 µL/min reached 65%. In contrast, the mixing efficiency of the single-phase flow at the same inlet liquid flow rate was only 6.5%. It can be concluded that compared to single-phase flow in the straight channel, the mixing efficiency can be increased nine-fold by using the Taylor flow under the same inlet total liquid flow rate.

### 3.3. Effect of Liquid Flow Rate on Mixing

**Figure 6:**
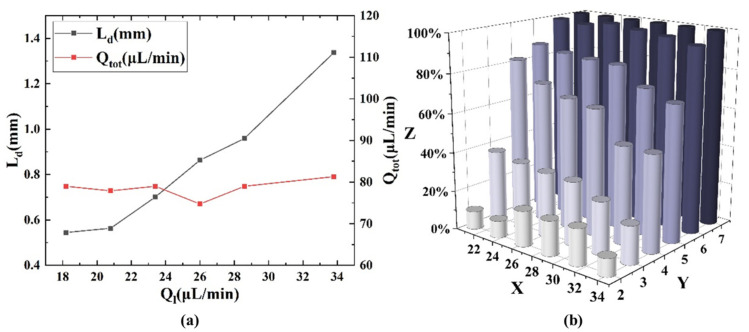
(**a**) Variation in droplet length and total flow rate with inlet total liquid flow rate. The droplet length decreased with increasing liquid flow rate, while total flow rate remained essentially unchanged. (**b**) Effect of different inlet liquid flow rates on mixing efficiency. The mixing efficiency increased with the increase in mixing distance (i.e., the increase in the number of observation point) and decreased with the increase in liquid flow rate, where X axis represented the total liquid flow rate (µL/min), Y axis represented the observation point, and Z axis represented the mixing efficiency.

With the inlet gas pressure was held constant at 110 mbar, under different total liquid flow rates of 18.2 µL/min, 20.8 µL/min, 23.4 µL/min, 26 µL/min, 28.6 µL/min, 31.2 µL/min, and 33.8 µL/min, the droplet length decreased with increasing total liquid flow rate, while the total flow rate remained essentially unchanged, as depicted in Figure 6a. Therefore, the increase in liquid flow rate essentially resulted in an increase in droplet length, and its impact on total flow rate can be neglected. The mixing efficiency ($e_{mix}$) with respect to different inlet total liquid flow rates and observation points is shown in Figure 6b. It can be observed from the figure that the mixing efficiency increased with the increase in mixing distance (i.e., the increase in the number of observation points) at any total liquid flow rate, and mixing efficiency was generally higher at lower inlet liquid flow rates. In other words, larger droplet length corresponds to lower mixing efficiency at the same observation point. Droplet length has a significant impact on mixing because increasing the droplet length weakens the internal circulation within the droplet [27], thereby reducing mixing efficiency. Therefore, long droplet requires a long mixing distance, and short droplets are better suited for short-distance mixing in serpentine channels.

Shorter droplets and higher flow rates enhance mixing by intensifying internal vortices and chaotic advection. Smaller droplets exhibit stronger curvature-induced vortex asymmetry in serpentine channels, while increased flow rates amplify flow reversals, both promoting fluid stretching/folding. This aligns with our 9× mixing improvement (Figure 5) and matches theoretical scaling laws [30,34]. As shown in Figure 6b, it was noteworthy that at observation point 2, the degree of mixing for droplets generated at both higher and lower inlet liquid flow rates was lower than that for droplets generated at intermediate inlet liquid flow rates. According to Che et al. [29], this is due to the influence of the droplet length on the internal flow patterns of the droplets. When the droplet is relatively short, it is influenced by the front and rear interfaces, resulting in lower mixing efficiency. Conversely, when the droplet is longer, it has a longer circulation path, requiring more time to complete one cycle, resulting in relatively lower mixing efficiency at the same time. The intermediate length had neither significant interface effects nor a long circulation path, so it had higher initial mixing efficiency. However, with the increase in mixing distance, the advantage of shorter droplets gradually became apparent, and it can be observed that after observation point 2, shorter droplets (i.e., lower inlet liquid flow rates) can result in more rapid mixing.

### 3.4. Effect of Gas Pressure on Mixing

**Figure 7:**
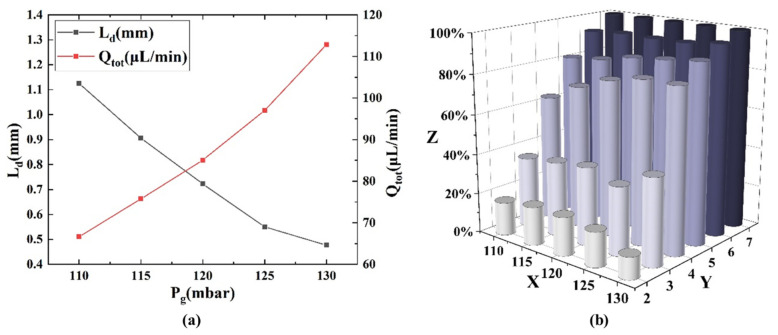
(**a**) Variation in droplet length and total flow rate with inlet gas pressure. The length of the droplet decreased as gas pressure increased, while the total flow rate increased. (**b**) Effect of different inlet gas pressures on mixing efficiency. The mixing efficiency increased with the increase in mixing distance (i.e., the increase in the number of observation points) and gas pressure, where the X axis represented the gas pressure (Pa), the Y axis represented the observation point, and the Z axis represented the mixing efficiency.

With the inlet total liquid flow rate maintained at 26 μL/min, various inlet gas pressures, including 110 mbar, 115 mbar, 120 mbar, 125 mbar, and 130 mbar, were examined. The results showed that changes in inlet gas pressure led to variations in both droplet length and total flow rate, as depicted in Figure 7a. All these sets of inlet conditions fell within the range for Taylor flow generation (Stage III in Figure 4). Droplet length decreased as the inlet gas pressure increased, while the total flow rate increased. It can be observed that inlet gas pressure contributed to the changes in both total flow rate and droplet length, with total flow rate primarily affected by inlet gas pressure. The mixing efficiency of the droplet at different observation points and under various inlet gas pressures was recorded, as shown in Figure 7b. The mixing efficiency of the droplet increased with increasing mixing distance (i.e., the increase in the number of observation points) and inlet gas pressure. This is because higher inlet gas pressure leads to shorter droplets and higher total flow rate, both of which enhance internal circulation and achieve mixing at a shorter distance. With the increase in the total flow rate, the droplet movement was accelerated, and the required mixing time decreased for the same mixing distance.

### 3.5. Evaluation of the Micromixer Designed in This Paper

While achieving comparable mixing efficiency (≥95%) to complex 3D mixers [24,25], our design reduces fabrication complexity through two key innovations: 1. Single-layer serpentine optimization. The curvature ratio (Rc/Dh = 2) generates equivalent Dean vortices to multi-layer designs [19] while maintaining planar fabrication [24]. 2. Self-aligning droplet generation. The 30° Y-junction (Figure 1a) eliminates the need for flow-focusing nozzles [25], reducing pressure fluctuations by 40% (∆P = 12 ± 3 kPa vs. 20 ± 5 kPa). Table 1 shows the comparison between this designed micromixer and some previous classical micromixers reported. The key parameters and mixing performance of micromixers are displayed. Our work departs from classic designs in three key aspects: (i) Systematic mapping of inlet conditions: We experimentally correlate gas pressure (110–130 mbar) and liquid flow rates (18.2–33.8 µL/min) to droplet length and mixing efficiency (Figure 6 and Figure 7), providing actionable guidelines for low-Re applications—a gap noted in recent reviews [23]. (ii) Manufacturing scalability: Unlike silicon/glass-based devices [22,25], our PMMA chip uses plasma bonding, enabling cost-effective, clog-resistant fabrication compatible with disposable LOC systems [9]. (iii) Performance benchmarking: Compared to recent alternatives [24], our mixer reduces mixing length by 33% (2 cm vs. 3 cm) and time by 58% (0.5–0.8 s vs. 1.2 s) at similar Re ranges (Table 1).

## 4. Conclusions

The achieved specifications meet WHO ASSURED standards for point-of-care devices, particularly the ‘Rapid’ and ‘Equipment-free’ requirements through sub-second mixing in disposable chips. To achieve rapid mixing of liquid reagents on a chip, we designed a droplet micromixer based on Taylor flow and chaotic convection. An experimental setup was constructed to validate the mixing performance of the mixer. Initially, we determined the range of inlet conditions that can generate Taylor flow and compared the mixing efficiency of the Taylor flow with single-phase flow under the same experimental conditions. Subsequently, we investigated the impact of inlet liquid flow rates and gas pressure on droplet length, total flow rate, and mixing efficiency.

This work prioritizes empirical validation of the Taylor flow-enhanced mixing under practical operating conditions (e.g., gas pressure, flow rates), addressing a gap in experimental data for low-Re applications. Prior studies [34] have extensively modeled droplet mixing dynamics, confirming that vortex evolution and mixing scales correlate predictably with droplet length and Re—parameters we systematically tested here. Our experimental results (e.g., nine-fold efficiency gain in Figure 5) empirically validate these established mechanisms.

According to the experimental results, both droplet length and total flow rate play crucial roles in determining the mixing performance within the droplet and are influenced by the inlet liquid flow rate and gas pressure. Shorter droplets and larger total flow rate enhance internal liquids’ mixing. Therefore, during the regulation process, it is advisable to adjust the inlet liquid flow rate and gas pressure to minimize droplet length while maximizing the total flow rate to facilitate mixing.

Our work bridges the gap between classical Taylor flow studies and contemporary microfluidic needs by demonstrating that simple geometries, when optimized for ultra-low Re regimes, can outperform complex modern designs. This is particularly relevant for applications where miniaturization, energy efficiency, and biocompatibility are prioritized over absolute throughput. We acknowledge the value of dimensionless analysis (e.g., capillary number effects) and plan to integrate simulations in future studies exploring broader parameter spaces.

## Figures and Tables

**Table 1:** Comparison of the mixing performance of this work and similar reported works.

Research	Channel Dimensions	Re	Mixing Efficiency	Mixing Length	Mixing Time	Key Limitation vs. Our Work
This work	300 × 150 µm	0.57–1.05	≥95%	<2 cm	0.5–0.8 s	N/A (baseline)
Fries et al. [22]	200 × 150 µm	11.6–22.6	≥95%	>12 cm	~0.8 s	High Re, long channel
El-Ali et al. [25]	400 × 300 µm	0.18–0.74	≥95%	<3 cm	1.2–4.8 s	Slower mixing
Pinho et al. [24]	250 × 150 µm	0.3–5.0	>90%	>3 cm	>1.2 s	Bi-phasic focus, longer mixing

## Data Availability

The original contributions presented in the study are included in the article, further inquiries can be directed to the corresponding author.

## Supplementary Materials

The following supporting information can be downloaded at: https://www.mdpi.com/article/10.3390/mi16060715/s1, Figure S1: The length of droplet measurement program; Figure S2: The grayscale standard deviation of droplet reading program.
