# Supplementary material for: Investigation of Efficient Mixing Enhancement in a Droplet Micromixer with Short Mixing Length at Low Reynolds Number
Source: Micromachines (Basel). 2025 Jun 16;16(6):715. doi: 10.3390/mi16060715 (PMC12195070; doi:10.3390/mi16060715)
Supplement: Supplementary file 1 [file micromachines-16-00715-s001.zip › micromachines-3639703-supplementary.pdf]

Measurement of droplet length

The original image captured by a high-speed camera contained both complete droplets generated by Taylor flow and part of the microchannels. To measure the length of droplet in the images, we used NI Vision Assistant in LabVIEW to write a program as shown in Figure S1 to measure the droplet length. The principle of this measurement program is to determine the droplet position according to the front and rear interfaces of the droplet and measure the droplet length along the channel. In the original image, a clear grayscale difference was observed between the liquid film and other regions, seeing as Figure S1 (a). Hence, grayscale segmentation was performed through 'Threshold' operation to extract the liquid film portion containing information about the droplet length, as shown in Figure S1 (b). Before 'Threshold' operation, the origin image was processed by 'Smoothing - Gaussian' operation and 'Convolution - Highlight Details' operation to make the edges of the image extracted smoother. Subsequently, operations such as 'Proper Open', 'Fill holes' and 'Remove small objects' were applied to enhance the integrity of the liquid film's edges and eliminate interference from small particles, seeing as Figure S1 (c). Following these steps, the 'Image Calibration' operation was carried out, and the scale developed in advance was imported into the program. Finally, the 'Clamp' operation was employed to measure the contour of the target, as shown in Figure S1 (d), and the droplet length (L_d) can be obtained. Sometimes the microchannels in the obtained images were not horizontal, and the droplet length obtained using 'Clamp' was not accurate, in which case the point-to-point distance measurement using the "Caliper" operation can ensure measurement accuracy, seeing as Figure S1 (e).

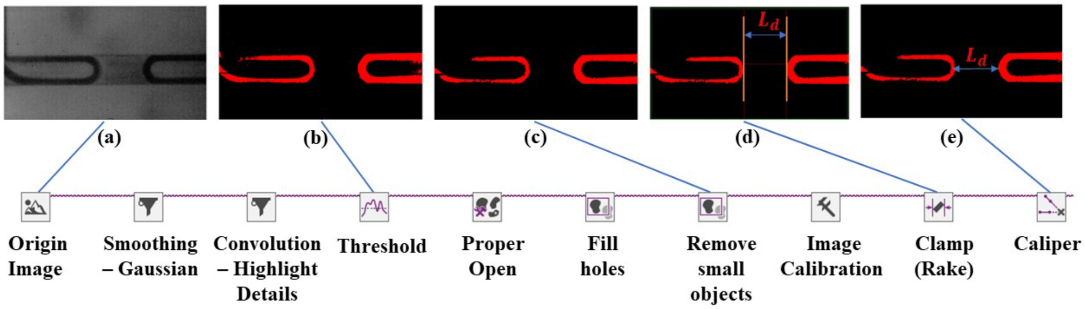

Figure S1. The length of droplet measurement program.

Measurement of droplet grayscale standard deviation

The grayscale standard deviation inside the droplet serves as an indicator of its mixing efficiency. We used NI Vision Assistant in LabVIEW to write a program as shown in Fig. S 2 to measure the grayscale standard deviation of droplet. Firstly, the "Smoothing - Gaussian" operation, "Convolution - Highlight Details" operation and "Proper Close" operation were carried out on the original image to remove the noise interference and dark spots in the image and smooth the region boundary. The processed image was named image 1, as shown in Fig. S 2 (b). After these operations, the image 1 was performed the ‘Image Buffer’ operation to store for subsequent droplet grayscale reading. Subsequently, the program performed the first round of ‘Threshold’ operation on the image to achieve grayscale segmentation, as shown in Fig. S 2 (c). Next, further operation was applied to extract the parts of the droplet, liquid film and microchannel in the image, requiring ‘Fill holes’ and ‘Remove small objects’ operations. The processed image is shown in Fig. S 2 (d). The ‘Image Buffer’ operation was carried out to retrieve the image 1 stored before. Following this, the ‘Operators-Mask’ operation was performed on the grayscale image to obtain the grayscale image containing only droplet, liquid film and microchannels, seeing as Fig. S 2 (e). Then, a series of operations, including ‘Threshold’, ‘Fill holes’, ‘Remove small objects’ and ‘Smoothing-Median’, were applied to extract the droplet in the image, as shown in Fig. S 2 (f) and (g). Finally, ‘Image Buffer’ was carried out to retrieve the image 1 and ‘Operators-Mask’ was carried out to

obtain the grayscale image containing only the droplet, seeing as Fig. S 2 (h). The grayscale standard deviation of this image can be directly read by 'Histogram' operation as 15.35, seeing as Figure S2 (i). In this study, the mixing efficiency in the droplet can be calculated by:

$$e_{mix} = \left(1 - \frac{\sigma}{\sigma_0}\right) \times 100\%$$

where σ_0 is the standard deviation of the grayscale value in the initial unmixed state, the fixed value of which is 80. At this time, the value of σ is 15.35, the mixing efficiency (e_{mix}) can be calculated as 80.8%.

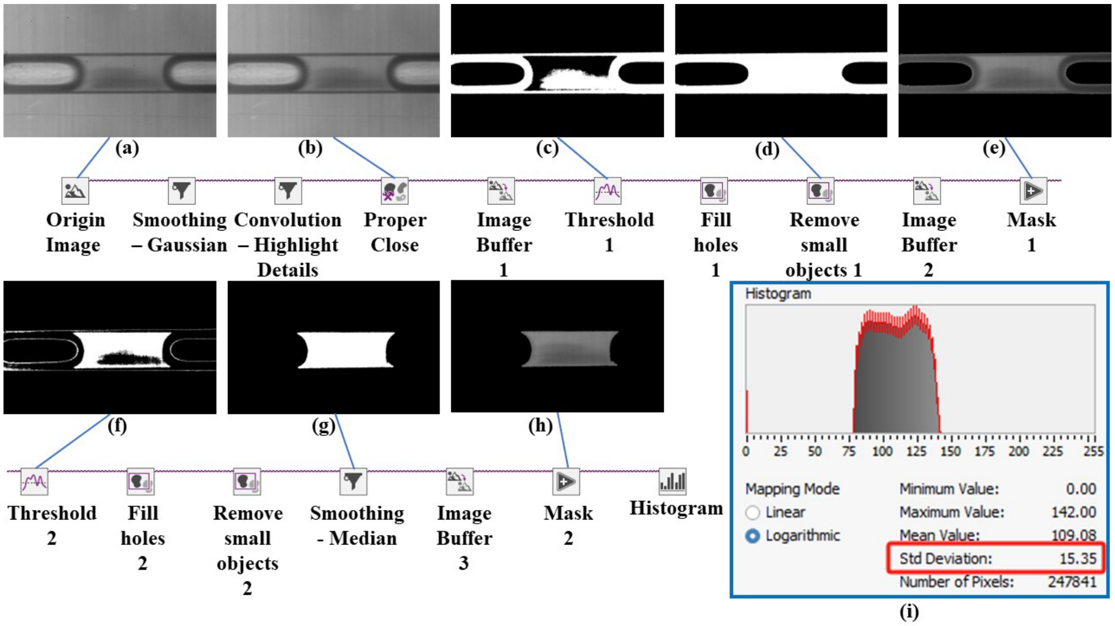

Figure S2 The grayscale standard deviation of droplet reading program.
